# Supplementary material for: Co-infections and risk factors of Toxoplasma gondii infection among pregnant women in Ghana: A facility-based cross-sectional study
Source: PLoS One. 2025 May 28;20(5):e0324950. doi: 10.1371/journal.pone.0324950 (PMC12118895; doi:10.1371/journal.pone.0324950)
Supplement: Attachment — Submitted filename: Comments to authors_PONE-D-25-00942R1.pdf [file pone.0324950.s002.pdf]

PONE-D-25-00942R1

Title: Co-infections and Risk Factors of Toxoplasma gondii Infection Among Pregnant Women in Ghana: A Facility-Based Cross-Sectional Study

Comments to authors

I appreciate the authors for point-by-point answer and response to the reviewer comment and suggestion. But some responses were still being missed.

Comment 1: The authors responded that they have revised all instances in the text to consistently use "co-infection" when referring to additional infections with HIV, HBV, and syphilis. However, usage of congenital infection is still occurred. Please recheck throughout the manuscript.

Example: Line No. 11: "miscarriage rates are uncertain and could be linked to toxoplasmosis and other congenital infections".

Line No. 216: "Prevalence of Congenital Infections"

Line No. 221: "Table 1: Prevalence of some Congenital infections Screened at ANC"

Comment 2: The authors responded that Variables are now described consistently as "socio-demographic," "environmental," and "obstetric factors" across both the methods and results sections. Can you explain about "Transmission Dynamics" in Line No. 258?

Comment 3: The authors responded that the data were verified. Corrections were made to align frequencies and percentages with the actual number of T. gondii positive cases (100). However, it can be noted that in table 4, there are (51+27+14 = 92) *T. gondii* positive in the variable of "type of animals". Other variables also differ with *T. gondii* positive cases (n=100). Can you explain about these variations?

Comment 4: Same as comment 3, in table 5, there are (1+70 = 71) *T. gondii* positive in the variable of "Child with hearing loss". "Miscarriage history" also differs with *T. gondii* positive cases (n=100). Can you explain about these variations?
