# Supplementary material for: Co-infections and risk factors of Toxoplasma gondii infection among pregnant women in Ghana: A facility-based cross-sectional study
Source: PLoS One. 2025 May 28;20(5):e0324950. doi: 10.1371/journal.pone.0324950 (PMC12118895; doi:10.1371/journal.pone.0324950)
Supplement: Attachment — Submitted filename: Response to Reviewers.docx [file pone.0324950.s003.docx]

**Rebuttal Letter**

**Assoah Ebenezer**
MPhil Biology (Parasitology)
Department of Biological Sciences
Akenten Appiah-Menka University of Skills Training and Entrepreneurial Development
Mampong Campus, Ghana
Email: assoahebenezer16@gmail.com ; Call: +233 205269064; WhatsApp: +233 543967381

**Date:** [19-04-2025]

**Editorial Board**
PLOS ONE

Dear Editors,

I am pleased to resubmit our revised manuscript, originally titled “**The Burden and Transmission Dynamics of Toxoplasmosis in Relation to Congenital Diseases among Pregnant Women in Ghana” (Manuscript ID: [PONE-D-25-00942])** and now titled **“Co-infections and Risk Factors of *Toxoplasma gondii* Infection Among Pregnant Women in Ghana: A Facility-Based Cross-Sectional Study,”** for consideration for publication in *PLOS ONE*. We are grateful for the constructive and astute feedback provided by both reviewers and the editor. In response, we have carefully revised the manuscript and addressed all the comments in detail. Please find below a summary of the major changes made:

- **Title Revision**: Based on Reviewers’ Suggestion and Comment, we have revised the title to better reflect the scope of the study, including co-infections and the risk factors associated with *Toxoplasma gondii* infection.
- **Abstract Revision**: The conclusion of the abstract has been revised to include practical preventive and promotive measures, as recommended in Reviewers’ Comment. This change highlights the significance of the findings for maternal and public health interventions.
- **Figure Removal**: As per Reviewers’ and editor Comment, and in line with PLOS ONE’s open-access and copyright policy, we have removed the figure 2 that was originally created using Google Maps, which is not compatible with the CC BY 4.0 license and figure 1 which confuses with conceptual framework.
- **In-text Citation Addition**: In response to Reviewers’ Comment, we have included the appropriate citation for the toxoplasmosis prevalence rate (92%) used in the sample size estimation.
- **Results Section Reorganization**: The presentation of results has been modified to follow a logical and consistent sequence, in line with Reviewers’ Comments. This enhances readability and coherence.
- **Co-infection and Model Fitness**: In response to Reviewers’ Comments, we have added a calculation of co-infection rates and included a detailed explanation of the regression model fitness, thereby improving the statistical rigor of the manuscript.
- **Ethical Considerations**: The type of informed consent obtained from participants has now been clearly stated under the section titled “Ethical Review and Clearance,” ensuring transparency in ethical compliance.
- **Study Design and Generalizability**: We appreciate Reviewers’ critical observations concerning the study design and potential limitations. We acknowledge that recruiting participants solely from health facilities may introduce selection bias and that the sample size may not fully represent the broader pregnant population. These limitations are now clearly addressed in the “Limitations of the Study” section. Additionally, to improve representativeness, data were collected over a ten-month period (15th August 2023 to 15th May 2024) across six health facilities within the Asante Mampong Municipality.
- **Conclusion Enhancement**: Following Reviewers’ Comment, the conclusion has been revised to integrate actionable recommendations for preventive and promotive maternal health interventions, thereby strengthening the practical relevance of our findings.
- **Funding Statement Removal**: We have removed the funding details from the manuscript text, as per PLOS ONE's policy, and ensured this information is included only in the online submission form.

We believe these revisions have significantly improved the clarity, depth, and utility of our manuscript. We appreciate the opportunity to address the reviewers’ concerns and are hopeful that our revised submission now meets the standards required for publication in *PLOS ONE*.

Thank you for your time and consideration.

Sincerely,
**Assoah Ebenezer**
(On behalf of all co-authors)
